# Prevalence and Risk Factors of Cervical Dysplasia among Human Immunodeficiency Virus Sero-Positive Females on Highly Active Antiretroviral Therapy in Enugu, Southeastern, Nigeria

**DOI:** 10.31557/APJCP.2019.20.10.2987

**Published:** 2019

**Authors:** Cornelius Osinachi Ogu, Peter Uwadiegwu Achukwu, Peter Onubiwe Nkwo

**Affiliations:** 1 *Department of Medical Laboratory Sciences, Faculty of Health Sciences and Technology,*; 2 *Department of Obstetrics and Gynaecology, Faculty of Medical Sciences, College of Medicine, University of Nigeria Nsukka, Nigeria.*

**Keywords:** Cervical dysplasia, HIV sero-positive, Highly Active Antiretroviral Therapy, Risk factors

## Abstract

**Objective:**

Evaluation of prevalence and risk factors of cervical dysplasia among Human Immunodeficiency Virus sero-positive (HIV+ve) females on Highly Active Antiretroviral Therapy (HAART) attending HIV clinic at University of Nigeria Teaching Hospital (UNTH) Enugu, Southeastern, Nigeria.

**Methods:**

Structured questionnaire was used to obtain socio-demographic and risk factors data. Cervical specimens were collected from 105 HIV +ve females on HAART and 104 HIV seronegative (HIV–ve) females. Pap smears were collected using cytobrush and Ayre’s spatula in a secluded place. Smears were made on slides and placed in 95% ethyl alcohol for conventional Pap staining and the cytobrush washed into the preservative containers for later Immunocytochemistry staining. Blood samples were used for HIV screening. Immunocytochemistry activity using anti-P16INK4A was carried out on the Pap smears that were positive for cervical dysplasia.

**Results:**

Pap staining showed prevalence of cervical dysplasia among HIV+ve on HAART 19.05%, (ASCUS 14.29%, LSIL 3.81%, HSIL 0.95%) whereas HIV-ve was 6.73%, p = 0.008. Only the HSIL 0.95% was positive for P16INK4A. Odds ratios at 95% Confident Interval of the risk factors of cervical dysplasia were thus; HIV+ve, 3.26 (1.31-8.09), education less than secondary school 3.23 (1.25-8.37), polygamy 3.23 (1.25-8.37), smoking 1.36 (0.15-12.10), married 2.08 (0.43-2.31), grand multi gravidity 1.72 (0.72-4.11), grand multi parity 1.54 (0.66-3.61), positive history of sexually transmitted diseases 2.49 (1.06-5.80). Uptake of cervical cancer screening was low in both study groups, 7 (6.7%) among HIV+ve on HAART and 14 (13.5%) among HIV-ve females, P = 0.102.

**Conclusion:**

HAART had cytoprotective effect against cervical dysplasia in HIV+ve females, by reducing progression of ASCUS to LSIL, HSIL and cervical cancer. Progression from normal to ASCUS increased which could be due to latency or/and prolonged persistent high risk HPV and HIV infections, of the most sexually active age group before diagnosed of HIV.

## Introduction

Cervical cancer can be avoided if detected early enough and treated. Pre-cancerous lesions are detectable for ten years or greater, before cancer develops (Mutyaba et al., 2006). Cervical cancer is the commonest genital cancer worldwide and one of the leading causes of death from cancer among women in developing countries (World Health Organisation 2012). Rates are highest in Central America, Sub Saharan Africa and Melanesia (WHO 2012). In Nigeria, cervical cancer accounts for 59-77% of gyneacological cancer, (Gharoro 1999; Galadanci et al., 2003; Aboyeji et al., 2004; Kyari et al., 2004; Mohammed et al., 2005; Mutihir 2005).

UNAIDS, (2014), reported that Nigeria carries the second highest HIV/AIDS burden in the world, with 3.4 million people living with HIV by the end of 2014. The National Prevalence as at 2014 was 4.1%. HIV infection and cervical cancer disease are common in the most populous black nation in the world – Nigeria (WHO/ICO 2010). Enugu state has the highest prevalence of HIV infection in the southeastern region (FMOH 2006).

Cervical cancer became Acquired Immunodficiency Syndrome (AIDS) defining illness among women with HIV in 1993 (CDC, 1993). HIV+ve females have increased prevalence compared to general population with standardized incidence ratios (SIRs) of 4.2 to 8.9 (Chaturvedi et al., 2009). Prevalence of cervical dysplasia among HIV+ve women in Nigeria differ in different regions, 11-56%, (Chama et al., 2005; Arnolu et al., 2011; Dim et al., 2011; Lawal et al., 2017; Muhammad et al., 2017). Hence, by 2003, WHO recommended HAART for HIV+ve irrespective of the CD4+ cell count (WHO, 2004). Kaposi’s sarcoma and Non Hodgkin Lymphoma declined after introduction of HAART but cervical cancer reduction was marginal (Ghebre, 2017). The burden of HIV and cervical cancer diseases are concentrated in Sub Saharan Africa. Cervical cancer runs a more fulminant course in HIV+ve women (CDC, 1993).

However, the most important risk factor of cervical cancer is chronic persistent oncogenic Human Papillomavirus (HPV) infection. Women with HIV are more likely to have persistent HPV, common in low and middle income countries (LMIC) (Torre et al., 2015). Speculation that HIV infection and consequent immunodeficiency resulting from infection negatively affects HPV clearance (Hankins et al., 2000), consequently increases prevalence and incidence of cervical dysplasia and cervical cancer.

It was expected that introduction of HAART, which decreases viral load and improves CD+4 cell, would enhance clearance of HPV thereby reducing incidence and prevalence of cervical dysplasia. Paucity of report about cytoprotective effect of cervix by HAART exists in our region and Nigeria, hence this research studied the cytoprotective effects of HAART on the cervix of HIV+ve females in Enugu, Nigeria.

The aim of this study was therefore, to evaluate the prevalence and risk factors of cervical dysplasia among HIV+ve females on HAART who attend HIV clinic at UNTH, Enugu, southeastern, Nigeria.

## Materials and Methods

This was a cross sectional, comparative study. This study was conducted in Enugu state, Southeastern Nigeria at UNTH. UNTH is the largest tertiary hospital in the region and serve as major reference hospital. It has the largest HIV center in the region and serves the region and beyond.

Minimum sample size (n) was calculated using Pocock (1983) and Sealed Envelope Ltd. (2012) 

Percentage ‘success’ in control that is prevalence of abnormal cervical smear as studied in Enugu was 12.2% (Chukwuali et al., 2003)

Percentage ‘success’ in experimental group that is prevalence of cervical pre-cancer and cancer in the study population of HIV-positive women in Nigeria was 6%, (Ononogbu et al., 2013).

Sample size required for each group = 95

Adjustment for non compliance or cross over = 10% of 95 =9.5↑ 10

Total sample size = 210

Ethical approval document was obtained from the ethical committee on research, UNTH Enugu. (NHREC/05/01/2008B-FWA00002458-1RB00002323). Letters of introduction were written to HIV center and Family planning unit, Obstetrics and Gynaecology UNTH Enugu to enable us use their facilities for the research. Written and signed informed consents were obtained from each participant with assured confidentiality of results. Participants whose results were positive for cervical lesions were referred for further investigation and management at O and G department UNTH.

Participants included females aged between 18 – 65 years, married and single non virgins who consented. The research excluded pregnant women, cervical cancer patients, menstruating females, previous HPV testing and/or Pap smear of less than 6 months, past hysterectomy, treatment of pre-malignant or malignant disease and mentally unstable females.

Data were collected from July 2018 – January 2019. Data were collected using structured self and interviewer administered questionnaire. The questionnaire comprised of section –A: Socio-demographic characteristics, section-B: Risk factors of cervical dysplasia.

Sampling was non probability. The targeted were those females who attend HIV clinic at UNTH, Enugu, Gynaecology clinic, family planning clinic and staff of the hospital. Specimens were collected from participants, who consented and passed the inclusion criteria in a secluded area in the hospital. Samples were collected from 105 HIV+ve females on HAART who attend HIV clinic at UNTH and 104 HIV-ve females who served as control. Each participant had specific coded number.

Papanicolaou staining and Immunocytochemistry (ICC) staining for tumour marker P16INK4A using conversional Pap staining technique and thin preparation from liquid based cytology respectively were done. Specimens were collected from the endocervix and ectocervix using cytobrush and Ayre’s spatula for Pap smear and immunocytochemistry by trained nurses, Doctors and Medical Laboratory Scientists.

**Figure 1:**
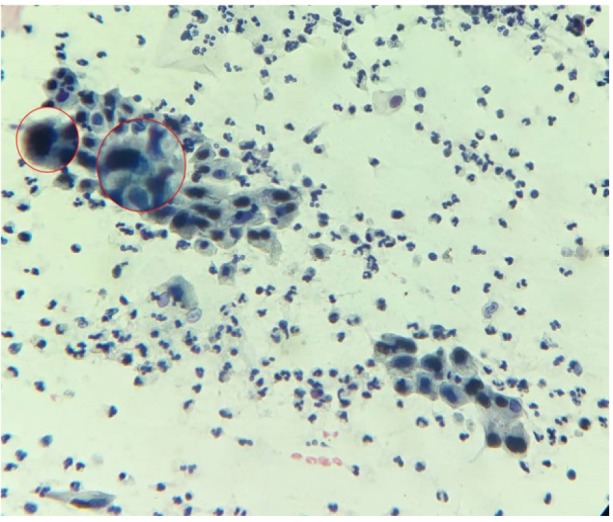
Shows the High-Grade Squamous Intraepithelial Lesion (HGSIL).

**Figure 2:**
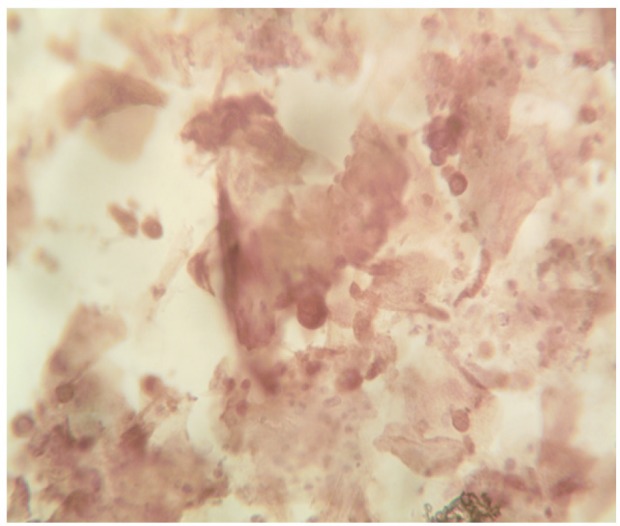
Shows Positive Immunocytochemistry (P16INKA4) Activity on the HGSIL Specimen

**Table 1:** Prevalence of Cervical Dysplasia between HIV+ve Females on HAART and HIV –ve Females

	Cervical dysplasia - positive				Cervical dysplasia -negative		
	ASCUS (percent)	LSIL (percent)	HSIL (percent)	TOTAL (percent)	Inflammatory (percent)	Normal (percent)	TOTAL
HIV +VE (105)	15 (14.29)	4 (3.81)	1 (0.95)	20 (19.05)	27 (25.71)	58 (55.23)	105
HIV –VE (104)	5 (4.81)	2 (1.92)	0	7 (6.73)	14 (13.46)	83 (79.81)	104
TOTAL (209)	20 (9.57)	6 (2.87)	1 (0.48)	27 (12.92)	41 (39.01)	141 (67.46)	209

ASCUS, Atypical Squamous Cell of Undetermined Significance; LSIL, Low-grade Squamous Intraepithelial Lesion; HSIL, High-grade Squamous Intraepithelial Lesion; HIV+ve, Human Immunodeficiency Virus sero-positive; HIV-ve, Human Immunodeficiency Virus sero-negative.

**Table 2:** Relationships between Risk Factors and Cervical Dysplasia Outcome among the Studied Population

Variables	Cervical Dysplasia		Odds Ratio (95% CI) P-value
	Positive [n=27 (percent)]	Negative [n=182 (percent)]	
Age (Years)			2.11 (0.60-7.38)
≥ 35	24 (88.9)	144 (79.1)	P = 0.24
< 35	3 (11.1)	38 (20.9)	
HIV Status			3.26 (1.31-8.09)
Seropositive on HAART	20 (74.1)	85 (46.7)	P = 0.01**
seronegative	7 (25.9)	97(53.3)	
Marital Status			2.08 (0.43-2.31)
Married	25 (92.6)	156 (85.7)	P = 0.34
single	2 (7.4)	26 (14.3)	
Educational Status			3.23 (1.25-8.37)
< Secondary	8 (29.6)	31 (17.0)	P = 0.12
≥ Secondary	19 (69.4)	151 (83)	
Marital Setting			3.23 (1.25-8.37)
Polygamy	8 (37)	22 (17)	P = 0.02*
Monogamy	17 (63)	151 (83)	
Occupation			0.69 (0.31-1.56)
Non civil servants	12 (44.4)	98 (53.8)	P = 0.36
Civil servants	15 (55.6)	84 (46.2)	
Smoking			1.36 (0.15-12.10)
Yes	1 (3.7)	5 (2.7)	P = 0.78
No	26 (96.3)	177 (97.3)	
Gravidity			1.72 (0.72-4.11)
>4	17 (63)	80 (44)	P = 0.21
≤ 4	10 (37.0)	81 (44.5)	
Parity			1.54 (0.66-3.61)
>4	11 (40.8)	41 (22.5)	P = 0.32
≤ 4	16 (59.3)	92 (50.5)	
Life Time Sex Partners			0.78 (0.33-1.84)
≥2	18 (66.7)	131 (72.0)	P = 0.57
1	9 (33.3)	51 (28.0)	
Coitarche			0.90 (0.23-4.87)
≤15	3 (11.1)	22 (12.1)	P = 0.88
>15	24 (88.9)	160 (87.9)	
Hormone Contraceptive USE			0.98 (0.61-1.57)
Yes			P = 0.96
No	7 (25.9)	48 (26.4)	
	20 (74.1)	134 (73.6)	
History of STDs/GTIs			2.49 (1.06-5.80)
Yes	18 (66.7)	81 (44.5)	P = 0.04*
No	9 (33.3)	101 (55.5)	

Pap smears were stained using Standard Operating Procedure for convetional Papanicolaou staining technique and sent to Cytopathologists/Histopathologists for microscopy and photomicrography. Report was according to Bethesda system of classification (2001) in Solomon et al., (2002).

Immunocytochemistry was carried out using mouse monoclonal anti-P16INK4A antibody. The analyses were done on only those Pap smear that had cervical dysplasia after Pap staining. Following standard protocol and company’s manual. Immunocytochemistry reagents were procured from Richard-Allan Scientific^®^, USA. Report was according to Han et al., (2008).

Blood samples were collected for HIV screening and confirmation using ELISA rapid test kits. HIV screening was carried out following the company’s instruction manuals. DETERMINE^®^ rapid kit.

The data obtained were analyzed using Statistical package for Social Sciences 20 and Microsoft® Excel statistical package at statistical significance level of 0.05. Chi-square (x^2^) was used to compare the results of the two groups of participants, Odds ratios, 95% Confident Interval was used to determine association between risk factor and cervical dysplasia. Student t-test for comparison of means, tables for data presentation, mean and standard deviations were used for continuous variables.

## Results

A total of 209 females actively participated in this research; 105 HIV+ve females on HAART and 104 HIV-ve females as control. The mean ages of the HIV+ve and -ve females were 42±4 years and 41.7±5.5 years respectively, P=0.65. HIV+ve on HAART had 43-47 years as modal group 29.5% (n=31) whereas age group 18-27 scored 0%. The modal age group for HIV-ve was 38-42 years 23.1% (n=24) and least age groups were 18-22, 23-27 and 63-67 1.9% (n=2). There was very highly significant difference in the marital status of the two groups of study p=0.00001; HIV+ve had more divorcees and widows than HIV-ve (8.6% vs 1.9%), (25.7% vs3.8%) respectively and 88.9% (n=24/27) of the HIV+ve widows testified that their husbands died of HIV infections.

Majority of the participants were from Enugu state; HIV-ve 56.7% (n=60), HIV+ve 76.2% (n=80). There was very highly significant difference in the educational status of the groups p=0.00001; more HIV-ve 79.8% attended higher institution than HIV+ve 33.3%. There was very highly significant difference in the occupational status of the two groups p=0.00001; HIV-ve had more civil servants compare to HIV+ve (63.5% vs 33.3%). Cervical Cancer Screening (CCS) uptake of the two groups, HIV-ve vs HIV+ve (13.5% vs 6.7%), p=0.10 though, both low. HPV vaccine receipt was low in both groups HIV+ve had 0% whereas HIV-ve had 4.8 (n=5), p=0.095. Details are on supplementary Table 1.

Prevalence of cervical dysplasia was 12.92% (n=27) among the participants which included 19.05% among HIV+ve females on HAART and 6.73% among HIV-ve females. There was statistically significant difference between the two groups, Odds Ratios at 95% Confidence Interval, OR (CI 95%) 3.26 (1.31-8.09) p=0.011. The commonest category of cervical dysplasia was Atypical Squamous Cell of Undetermined Significance (ASCUS); 14.29% in HIV+ve on HAART and 4.81% in HIV–ve females. Also the prevalence of each category was higher among HIV+ve females on HAART as shown on Table 1. Furthermore, the Pap smear staining results showed higher inflammation among HIV+ve on HAART 25.71% and 13.46% among HIV–ve, OR (CI 95%) 2.76 (1.33-5.71) p=0.0062. Atypical Squamous Cell cannot exclude High-Grade Squamous Intraepithelial Lesion (ASC-H) was 0.0%.

The Immunocytochemistry (ICC) analysis for tumour marker, P16INKA4 activity done on the Pap smears, positive for cervical dysplasia 12.92% (n=27) showed positive result only on the HSIL specimen 0.95% (1/105), which signified cervical cancer.

Majority of the risk factors of cervical dysplasia among the study groups showed positive association, OR (CI 95%): HIV+ve 3.26 (1.31-8.09) p=0.011; age above 35 years 2.11 (0.60-7.38); married 2.08 (0.43- 2.31); less than secondary school 2.05 (0.82-5.10); smoking 1.36 (0.15-12.10); gravidity above four 1.72 (0.72-4.11); parity above four 1.54 (0.66-3.61); polygamy 3.23 (1.25-8.37) p=0.02; history of STDs/GTIs 2.49 (1.06-5.80) p=0.04. details are showed on Table 2.

Our result showed that 80% of HIV+ve participants commenced HAART less than 1 year after diagnosis, while 91.4% have taken HAART for more than 1 year. Details are shown on supplementary Table 2.

## Discussion

Our study showed significantly higher prevalence of cervical dysplasia among HIV+ve females on HAART compared to HIV–ve females, which is consistent with previous studies about the relationship between HIV, HPV and cervical precancerous lesion (Blitz et al., 2013; Ezechi et al., 2014). From our study, the prevalence of cervical dysplasia among HIV+v on HAART and HIV–ve females were higher than report of Dim et al., (2011) who worked at the same centre but on HIV+ve women not on therapy; (HIV+ve; SIL 12.6%, ASCUS 1.3%, LGSIL 8.3%, HGSIL 3.3%, inflammation 14.0%, for HIV–ve; SIL 4.6%, ASCUS 4.4%, LGSIL 3.3%, HGSIL 1.3% inflammation 10.7). Some reports from other centers were also lower than ours (Anorlu et al., 2003, Durowade et al., 2012; Pimentel et al., 2013; Ezechi et al., 2014), but our report was lower than recent report of Kim et al., (2013); Lawal et al., (2017) in Abuja , Nigeria; Ugbuaja et al., (2017), which also worked on HIV+ve women not on therapy. The increase in ASCUS which was the contributor to higher prevalence in our study, could be due to risky sexual behavior among HIV+ve females on HAART as reported in previous studies that inception of HAART prompted HIV+ve to indulge in risky sexual behaviors (Chow et al., 2012; Ganesan et al., 2012). It could also be attributed to delay of the most sexually active group of females to avail themselves for voluntary counseling, testing and treatment, which prolongs the persistent infection of the cervix by oncogenic HPV and HIV infection. It could also be due to period of latency before cervical dysplasia manifested. Low perception of risk factors and lack of awareness about cervical cancer screening amongst women and challenges of availability to cervical cancer screening for early detection of disease have been reported amongst factors causing increasing incidence and mortality from cervical cancer in developing countries, (Ayinde et al., 2004, Dim et al., 2009; Ezechi et al., 2013).

However, from our study ASCUS was the actual contributor of higher prevalence of cervical dysplasia in our report compared to that of Dim et al., 2011 and supported by prior study of Blitz et al., (2013) which reported that rate of progression from normal to; ASCUS, LSIL HSIL and ICC were higher compared to rate of regression from these categories to normal in HIV +ve on HAART. They attributed this to presence of high risk HPV types and increased number of sexual partners, though generally progression and incidence of cervical dysplasia was reduced by HAART. Kim et al., (2013) during their study, at a stage termed “progression visit” noticed that 35% had progressed to ASCUS, 0.37% to ASC-H, 57% to LGSIL, 7% to HGSIL and 0.41% to cancer. From our study, other categories, LSIL and HSIL were of lower prevalence compared to Dim et al., (2011) and reports from outside our study center on HIV+ve not on therapy, hence one can say that HAART reduces progression of cervical dysplasia among HIV+ve females, which is consistent with some previous reports (Minkoff et al., 2010; Alder et al., 2012; Firnhaber et al., 2012; Kim et al., 2013; Blitz et al., 2013; Ezechi et al., 2014; Katz et al., 2016). Firnhaber et al., (2012) concluded that HAART use reduced the rate of incidence and progression of cervical lesions among HIV+ve females and was dependent on duration of regimen but Menon et al., (2017), reported in their systematic review of effects of HAART on cervical dysplasia in sub Saharan Africa, suggested that CD4+ cell count may have a more instrumental role in cervical oncogenesis or the integration of the latent reservoir throughout the body than either HAART use or the treatment duration on the prevalence of CIN 2 and CIN 3. Minkoff et al., (2010) concluded that effective and adherent HAART use was associated with a significantly reduced burden of HPV infection and Squamous Intraepithelial Lesions (SILs).

The ICC analysis for tumour marker, P16INKA4 activity showed positive result only on the HSIL specimen, which signified cervical cancer prevalence of 0.95%, which is similar as reported by Kim et al., (2012). ICC is the most sensitive technique to detect cancerous lesion, hence use of ICC will help to remove over diagnosis or under diagnosis of cervical cancer. ICC can be used to evaluate all cases of cervical dysplasia which can increase accuracy of diagnosis and management of cervical dysplasia.

From our study, the age distribution signified that early youthful ages were not captured, probably because they shy away from the tests or do not actually avail themselves for voluntary counseling, testing and treatment or don’t come for HAART. Studies on these younger ages are worth carried out because these are the most sexually active ages and are at higher risk of HIV infections (WHO, 2010) and Human papillomavirus infections. Human Papilomavirus is regarded as the primary etiologic agent of cervical cancer and transmission is basically by sexual intercourse, (Anorlu, 2008; Oakeshot et al., 2012). Absence or reduced number of these ages in this study could signify that most HIV positive female are exposed to high risk Human Papillomavirus and other risk factors of cervical dysplasia long time before screening, hence this could be one of the reasons HPV clearance is delayed among HIV positive female on HAART leading to failure in significant decrease or regression of cervical dysplasia among HIV positive females on HAART. Result of our research showed that more of the HIV+ve had first sexual intercourse between 10-19 years compared to HIV-ve females. We suggest that HPV vaccine, where and when available should be introduced as early as from 13 years and once a woman debut on sexual intercourse, to enhance protection against HPV and subsequent cervical dysplasia. 

Educational level showed that HIV–ve attended higher levels of education compared to the HIV+ve females. Our report was consistent with previous reports of Lawal et al., (2017) and Dim et al., (2011). Higher educational level among females can help to reduce HIV infection and cervical dysplasia. Lower educational status will leave them with low income jobs which can expose them to HIV infection and cervical dysplasia. Most of the HIV-ve engaged with civil service jobs as against HIV+ve females, who were engaged in trading, farming, self employment and house wives. HIV+ve females engaged in jobs that highly expose them to HIV infections due low income earning, Lawal et al., (2017) also had similar report from their research.

During our study, only 30% of HIV+ve offered free screening, accepted testing unlike in prior study by Odafe et al., (2013), who reported that 96.5% acceptance rate, similar with findings from studies in Kenya and Mozambique that reported acceptance rates of 87% and 86%, respectively, of cervical cancer screening using VIA technique, (Huchko et al., 2011; Audet et al., 2012). Nonetheless screening for cervical cancer in Nigeria remains poor, (Dim et al., 2009), this was also found in our research were uptake of cervical cancer screening and HPV vaccine among the participants were very low. This could be attributed to low perception and lack of awareness of the advantages of Cervical Cancer Screening (CCS) or because we don’t have organized CCS programme in Nigeria. Women who fail to go for CCS are at higher risk of cervical cancer.

Results of risk factors of cervical dysplasia between the studied groups showed statistically significant difference between the two groups in number of life sex partners, age at first sexual intercourse, and STDs/GTIs, p<0.05. From our research, associations showed that risk factors of cervical dysplasia included ages equal to or above 35 years, HIV+ve even on HAART, married women, educational status of less than secondary school, polygamous marriage, smoking, gravidity above 4, grand multi parity, positive history of STDs/GTIs. These were consistent with previous reports (Partridge et al., 2007; Oakeshott et al., 2012). Life sexual partner of more than two is not associated with cervical cancer unlike reported in previous works above.

In conclusion, HAART had cytoprotective effects against progression of ASCUS to LSIL, LSIL to HSIL and to Invasive Cervical Cancer but failed to prove so from normal cervix to ASCUS in HIV+ve females in Enugu, Nigeria. Failure of the most sexually active females to be captured in this research could be one of the reasons for the increased progression from normal to ASCUS and reduced regression of cervical dysplasia. Immunocytochemistry can be of great advantage in the accurate diagnosis of cervical cancer. Cervical Cancer Screening should be introduced as routine investigation for HIV+ve females in Enugu, Nigeria. Effective cervical cancer screening and HPV vaccination programme will be of great advantage in our region.

## Approval

The research was part of an approved thesis by School of Post Graduate Studies and Department of Medical Laboratory Sciences University of Nigeria Nsukka.

Research was not approved by any Scientific body.
